# Mental body representations of women with tattoos in emerging adulthood — a cluster analysis

**DOI:** 10.1007/s00737-023-01326-z

**Published:** 2023-06-01

**Authors:** Klaudia Jabłońska, Beata Mirucka

**Affiliations:** Department of Psychotherapy and Health Psychology, Institute of Psychology, The John Paul II Catholic University of Lublin, Al. Racławickie 14, 20-950 Lublin, Poland

**Keywords:** Mental body representations, Embodiment, Body modification, Tattoo

## Abstract

Tattoo is a permanent body modification relevant to women’s bodily experiences. Previous studies on the population of tattooers have focused mainly on searching for negative or positive aspects of tattooing, mainly in the field of self-esteem. The aim of this study was to explore the mental body representations of tattooed women during emerging adulthood. Three hundred and twenty-seven women with tattoos, aged 18 to 25 (*M* = 21.48; *SD* = 2.05), participated in the study. In the study, we adopted a quantitative research project; the data for analysis was obtained from questionnaires that were completed online between January and March 2021. Women with tattoos completed a survey containing demographic questions, questions regarding their tattoos, the Battery of Tests of Body Self Representations, and the Rosenberg Self-Esteem Scale. Based on that a cluster analysis, we have discovered three types of mental body representations that may characterize them: (1) unstable, (2) disordered, and (3) integrated. The study showed that having a tattoo is associated with experiencing carnality in a specific way.

**Supplementary Information:**

The online version contains supplementary material available at 10.1007/s00737-023-01326-z.

## Introduction

Tattooing is becoming more and more normative in the statistical sense as a way of modifying one’s body. In the past, the tattoo was found in distant cultural circles, where it usually had a ritual function. Through travellers and sailors, it was also spread to Europe, where it was initially associated with specific social groups until modern times, when people of different social statuses and genders began to opt for it. Tattooing is nowadays a widespread phenomenon, especially in the population of people falling within the age range of 18–26 years, the so-called emerging adulthood (Arnett 2000).

The developmental period called emerging adulthood is a stage distinguished by relative independence from social roles, on one hand, not a childhood in which there is dependence on adults, but not an adulthood that requires taking on specific life roles (Arnett 2000). Emerging adulthood is a moment of searching for one’s own identity, which includes experiencing one’s bodily self. The largest number of people who decide to get a tattoo is in this developmental period (Heywood et al. 2012); in one study, it was proved that almost half of the respondents (44%) got their first tattoo at the age of 18 (Alter-Muri 2020). It is also important that men decide to get tattoos more often than women (Lahousen et al. 2019), which may be related to a worse social evaluation of women with tattoos (Swami and Furnham 2007).

Research indicates that people with tattoos are perceived worse than those without tattoos (Forbes 2001), and women with tattoos are particularly vulnerable to worse perceptions (Swami and Furnham 2007). Because the female body is subject to constant appraisal from the environment and is subject to self-objectification Fredrickson and Roberts 1997), it is possible that a socially unacceptable form of body modification may be relevant to women’s experience of themselves.

Still, many young women decide to permanently change their bodies. Tattooing is used to decorate vs cover one’s body, which presumably becomes an important factor in the formation of one’s sense of self, one’s self-worth, and, as a further consequence, one’s personal, gender, and bodily identity. Tattooing and wearing tattoos as a visual aspect of self-creation are not without significance for the psychological functioning of the subject.

The involvement of the body in the tattooing process seems not to be indifferent to the experience of the embodiment of self. Mental body representations give information about the level of experiencing one’s carnality (Mirucka 2018). It seems that those involved in the tattooing process may experience their body in a specific way.

## Tattooing phenomenon

Body modification practices began to gain popularity in the late twentieth century (Ory 2020). One of such modifications is the tattoo, which we can define as a “marking on one’s skin, intentionally produced via one of many available techniques, which may perform various functions and have various meanings to its wearer” (Snopek 2018, p. 18). The process of tattooing involves a substantial alteration of the human body as the ink is inserted into the skin, usually the dermis layer, which had previously been punctured or cut, in order to create a desired image. A permanent alteration of an individual’s skin presumably has bearing on their psyche, considering that body experience is crucial to the formation process of the self (Damasio 2000; James 2002; Mirucka 2018).

The phenomenon of tattooing has been investigated throughout history, mainly by archeologists, anthropologists, sociologists, and pedagogues (Deter-Wolf et al. 2016; Kosut 2006; Samadelli et al. 2015; Snopek 2018), whose research helped create the initial classifications of tattoos according to their function and their wearer’s motivations. Within the realm of psychology, the initial research was primarily concerned with the relationship between tattooing and psychopathology. Tattoos were linked with personality disorders and drug use (Buhrich and Morris 1982; Gittleson et al. 1969; Raspa and Cusack 1990). They were classified as a form of nonsuicidal self-injury (Favazza and Rosenthal 1993; Favazza 1998, 2006). Modern scholars associate wearing tattoos not only with greater nonconformity (Forbes 2001, Nathanson et al. 2006), sensation seeking (Hong and Lee 2017; Roberti et al. 2004; Stirn et al. 2006), high-risk behavior (Drews et al. 2000; Hong and Lee 2017; Swami et al. 2016), drug use (Drews et al. 2000; Forbes 2001, Kertzman et al. 2019; Nathanson et al. 2006), but also with a high degree of self-awareness (Cipolletta et al. 2010; Mun et al. 2012). Still, the connection between tattooing (in the form of a permanent alteration of one’s external appearance) and mental body representations remains to be sufficiently explored.

## Tattoo and body

Tattoo is usually seen either as a decoration of the body (Craik 1994) or as a painful and permanent form of body modification (Sweetman 1999). These two — seemingly opposing — perceptions of the phenomenon paved the way for two distinct paths of research. The first path, which we will call “aesthetic,” focused on the self-appearance of tattooed individuals. Part of this research was concerned with self-esteem, which, in some publications, was shown to be much lower in tattooed individuals (Farrow et al. 1991; Raspa and Cusack 1990; Swami 2011); however, it was demonstrated that getting a tattoo could actually help boost one’s self-confidence, which was still evident in subjects three weeks after the procedure. Some works suggest the contrary, showing no difference in self-esteem between tattooed and nontattooed individuals (Deschesnes et al. 2006; Frederick and Bradley 2000).

## Self-esteem

Self-esteem, however, is not directly related to embodiment and, as such, cannot be used to effectively gauge how an individual experiences their own body. Nonetheless, some works treat body image as a separate variable. Kertzman et al. (2019) investigated the differences in self-esteem between women with and without tattoos. They determined that tattooed women displayed a greater difference between their real and ideal self, which was interpreted as evidence of their low self-esteem. The study did not find significant differences in body image between the two studied groups. Other researchers argued that tattooing is not so much associated with low self-esteem as it is with a negative perception of one’s own body (Carroll and Anderson 2002). Swami (2011) observed that individuals exhibited elevated body satisfaction, self-esteem, and a sense of uniqueness after getting a tattoo. This effect, while evident, was found to be rather short-lasting in women, who, when compared to male subjects, reported higher social anxiety surrounding their appearance 3 weeks after the procedure. Such attitude was likely related to their distress over being negatively evaluated by others, as tattoos are generally found to be less socially acceptable on women than men (Armstrong 1991; Armstrong et al. 2008; Braunberger 2000; Doherty 1998; Hawkes et al. 2004; Swami and Furnham 2007).

## Regulatory tattoo function

The second path of research is labeled “regulatory,” which ascribed self-harm characteristics to tattooing. Such an approach can be found in the works of Favazza (Favazza and Rosenthal 1993; Favazza 1998; 2006), who viewed the tattoo as a form of nonsuicidal self-injury. Thus, tattoos, in a manner similar to self-harm, are manifestations of affect regulation. According to this theory, an individual, through the act of tattooing, diverts their own attention from psychological discomfort (the overwhelming emotions) to somatic discomfort (bearable physical pain). Simultaneously, the very same act of tattooing might constitute an attempt to feel more alive when one feels dead inside owing to a limited internal experience (Kinecka 2006; Mirucka and Sakson-Obada 2013). Multiple studies demonstrated a link between tattooing and psychopathology, including self-destructive behavior (Kim 1991; Raspa and Cusack 1990; Roberts and Ryan 2002; Stirn and Hinz 2008; Wycisk 2004; 2001). Some research also associates body modification with psychological trauma (Atkinson 2002; Romans et al. 1998; Stirn and Möller 2013). Tattoos could help regulate trauma-related dissociation, a state of mental “death” — and loss of connection to one’s own body (Mirucka 2018; Sakson-Obada 2009). The abovementioned research shows that tattooing remains an elusive topic and that psychological mechanisms associated with it might constitute an important part of the body experience.

## Women with tattoos

Gender has a significant role in bodily experience. Women’s bodies are more often subject to social evaluation than men’s and are often self-objectified. Women perceive their bodies in the way the social environment does and try to match social standards of appearance (Fredrickson and Roberts 1997). Swami and Furnham (2007) showed that women with tattoos are perceived as less attractive, alcohol abusers, and sexually promiscuous. Research on body perceptions with tattoos was also conducted by Hawkes et al. (2004). The researchers concluded that women with tattoos are perceived worse than those without, regardless of the size and visibility of their tattoos. Currently, an increasing number of women are opting for tattoos, which may indicate that tattooing may represent an attempt to regain control of one’s own body and create it according to one’s own rules. It is not clear how tattoos affect women’s bodily experience. On the one hand, they may cause a sense of agency and overcome empowerment; on the other hand, once tattooed and exposed to social judgment, they may worsen the way one experiences one’s body.

## Body mental representations

One of the theories outlining the relationship between embodiment and shaping of personal identity is found in Beata Mirucka’s Embodied Subject Model (Mirucka 2018). According to this theory, the complex sphere of human embodiment can be divided into three levels: first — neuronal maps; second — mental body representations; and third — the sense of self.

The neuronal level comprises the internal mechanisms of body self-perception. Among them, we find the following: interoception (the reception of internal body stimuli), proprioception (reception of stimuli associated with our body’s movement and position in space), and exteroception (reception of stimuli originating from outside the body, upon coming into physical contact with a particular object). These mechanisms allow the human brain to create a neuronal map of their body, which in turn constitutes a foundation for the next level of bodily experience.

The second level of embodiment can be divided into three categories of mental body representations: (1) body image (perceptual body image, together with emotions and beliefs associated with the body (Gallagher 2005), (2) body schema (allows the control and movement of the body, creates a sense of bodily form), and (3) body sense (e.g., the complete experience of one’s physical condition).

The third, so-called self-identity level, is experienced the most consciously, and it is at this level that the body becomes part of the self. The body identity awareness on the highest level of bodily development is reflected in the constellation of senses: sense of self-existence, sense of continuity of the self through space and time, sense of internal wholeness, sense of being separate and aware of one’s body’s boundaries, acceptance of the self as a being, sense of agency, sense of control over one’s own body, and psychophysical integrity (Mirucka 2018).

There is a lack of research in the literature that addresses the impact of body modification in the form of tattooing on the way people experience their bodies. The few publications examining the relationship between tattooing and the body have focused on variables such as the bodily expression of uniqueness (Tiggemann and Hopkins 2011; Tiggemann and Golder 2006), body appreciation, appearance investment, self-ascribed uniqueness, self-esteem (Swami 2011), attractiveness (Drews et al. 2000), and body investment (Carroll and Anderson 2002). One study attempted to test for differences in body image between women with and without tattoos, but no significant differences were detected (Kertzman et al. 2019).

To the best of our knowledge, no such research has been conducted to date that takes as its aim the exploration of the quality and constellation of mental representations of the body (body image, body schema, and sense of body) in people with tattoos. Therefore, in identifying this lack, we decided to undertake the task of answering an important research question: How do women with tattoos in emerging adulthood experience their bodies? Do women with tattoos present one typical way of experiencing their bodies (arrangement of body representations), or are they characterized by several typical constellations of the three main body representations: body image, body schema, and body sense?

As the research problems posed are phenomenological in nature, the way to address them will be to (1) identify the phenomenon itself — the way women with tattoos experience their bodies and then (2) describe it in detail. This preliminary and at the same time necessary research stage (the so-called phenomenological one) seems to be very important, as its successful implementation will enable the transition to the next, more complex research on the tattoo phenomenon: correlational or experimental.

## Methods

### Design

We first reviewed the literature on body tattooing and the psychological variables studied in this context. We discovered a deficit in the area of research on the corporeality of women tattooing and decided to explore this area. Based on the current literature, we hypothesized that women with tattoos would differ in their experience of the body due to the system of psychological representations of the body. In the study, we adopted a quantitative research project; the data for analysis was obtained from questionnaires that were completed online between January and March 2021. Inclusion criteria for the study were as follows: age 18–25 years, female gender, and having at least one tattoo on the body. Exclusion criteria were the absence of tattoos, age over 25 years, and gender other than female. We calculated the power of the sample size (post hoc) using the G*Power software (Kang 2021): 1 − *β* = 0.9862.

### Operational definition

Tattoo: A fixed mark on the body, with different meanings for its bearer, the making of which involves the insertion of pigment under the skin by means of a needle.

Emerging adulthood: The developmental period in which individuals find themselves between the ages of 18 and 25 (Arnett 2000).

Body mental representation: As conceptualized by Mirucka (Mirucka 2018), these are body image, body schema and body sense, and their level indicating how a person’s corporeality is experienced.

### Measures

To conduct the study, we compiled the following methods to test the variables: (1) The Battery of Tests of the Body Self-Representations (BT-BSR; Mirucka 2018); (2) Rosenberg’s Self-Esteem Scale (SES; Rosenberg 1965); (3) the qualitative method “My tattoos,” created for this study; (4) Survey containing demographic questions and questions regarding tattoos.

#### The Battery of tests of the body self-representations

The utilized research tool consists of a set of three scales: the Scale of Body Schema (SBS), Scale of Body Image (SBI), and Scale of Body Awareness (SBA). SBS is used to investigate the body schema representations (e.g., aspects of control over one’s own body, predominantly linked to proprioception). The questionnaire contains six items (e.g., “I feel that my body limits me”; “My movements are full of grace and harmony”). High SBS score indicates a high degree of control over one’s own body as well as sense of their body being under their control and a part an integral part of the body self. The SBI scale, which primarily focuses on exteroception, is designed to gauge perceptions, beliefs and emotions directed at one’s own body. It includes 6 statements (e.g., “I look completely normal”; “There are parts of my body which I entirely refuse to accept”). High SBI score indicates confidence in one’s appearance and a positive perception of their body. Low scores, on the contrary, point at subject’s lack of acceptance of their own body and its negative effects on one’s social self. The third scale, SBA, which centers on interoceptive sensations, gauges the general sense of one’s physical condition, emotions directed at one’s body, as well as one’s physical needs. The SBA questionnaire contains 18 items (e.g., “I feel strong and healthy”; “I am ashamed of my body”; “I treat myself with body massages, baths, good meals, etc.”). High SBA scores indicate an ability to read one’s own body’s signals, capability to deal with strong emotions, and sufficient satisfaction of one’s own physical needs. Low scores link at low awareness of one’s own body signals, inability to deal with intense emotional states, and failure to satisfy one’s own physical needs. The responses were scaled using a 7-point Likert scale (from 1, Strongly disagree; 2, disagree; 3, mostly disagree; 4, Neither agree nor disagree; 5, Mostly agree; 6, Agree; 7, Strongly agree). *BT-BSR* has good psychometric properties. Cronbach’s alpha obtained during the development of scales are as follows: *α*_SBS_ = 0.75, *α*_SBI_ = 0.81, *α*_SBA_ = 0.86 (Mirucka 2018). All scales used in this study have also demonstrated high reliability. The Alpha values for the used scales measured: *α*_SBS_ = 0.78, *α*_SBI_ = 0.88, *α*_SBA_ = 0.88.

Rosenberg’s Self-Esteem Scale (SES) is a tool used for measuring self-esteem. The SES contains 10 items (e.g., “At times I think I am no good at all”; “I wish I could have more respect for myself”; “I take a positive attitude toward myself”), scaled using a 4-point Likert scale (from 1, Strongly Agree; 2, Agree; 3, Disagree; 4, Strongly Disagree). Self-esteem as measured by this tool is regarded as a relatively stable trait. It is a positive or negative attitude towards the self; self-esteem is therefore a global evaluation of the self. High scores suggest favorable self-perception, i.e., that person feels valuable; low scores, on the other hand, indicate dissatisfaction with oneself and a low sense of self-worth. The reliability coefficient (Cronbach’s alpha) of the SES sale used in this study is high (*α*_SES_ = 0.91).

*My tattoos* is a qualitative method we have designed to assess tattoos. It incorporates an illustration of a silhouette of a woman’s body, displayed from front and back. Both surfaces are divided into smaller areas forming individual parts of the body’s surface. The front silhouette encompasses head, neck, upper chest, chest, abdomen, upper arms, forearms, hands, pubic area, thighs and knees, tibia, and feet. The back silhouette is divided into the following: head, neck, upper back, lower back, upper arms, forearms, hands, buttocks, calves, and feet. This gives a total of 23 individual areas. The responders are tasked with marking the areas on the silhouettes that correspond to tattoos they themselves have. In addition, they are asked to provide the total number of tattoos they have and the time of getting their first tattoo.

### Study setting and participants

Three hundred and twenty-seven Polish women with tattoos, aged 18 to 25 (*M* = 21.48; *SD* = 2.05) participated in the study. We searched for respondents on social networking groups for tattooers. We posted on several of them about the research we were conducting, and 351 women signed up for the study. Respondents received a link to a series of questionnaires with information, about the anonymity of the study, the possibility to opt out at any time, and the possibility to ask the researchers questions, via email. The 24 completed surveys were not taken into account in the analyses due to incomplete completion of the questionnaires or too rapid completion times.

## Data analysis

SPSS version 27 was chosen as the tool used to perform the statistical data analysis for the purposes of this study. The other tools we used include k-means cluster analysis, analysis of variance (ANOVA), and Pearson correlation coefficient.

For the purposes of this study, the female participants were divided into four groups, roughly equal in size, based on the number of tattoos they had, as follows: single tattoo, two tattoos, three to five tattoos, and lastly, more than five tattoos (between 6 and 40 in our sample group) (see Table 1). In addition, the females were categorized according to the number of tattooed areas of the body. A total of four groups were distinguished (see Table 1). The fourth group consisted of participants with a minimum of six tattooed areas but could include as many as 23 areas; however, the highest number of tattooed areas among the participants was 15; this figure was reported by a single participant. Pearson correlation coefficient between the number of tattoos and tattooed areas of the body was very high (*r* = 0.88; *p* < 0.001). The majority of responders had had their tattoos for longer than 2 years (see Table 1).

**Table 1:** Number of tattoos, number of tattooed body areas, time of getting the first tattoo, education status, domicile, and occupational status

Variables	*n*	%
Number of tattoos		
1	86	26.3
2	81	24.8
3–5	86	26.3
> 6	74	22.6
Number of tattooed areas		
1	97	29.7
2	86	26.3
3–5	102	31.2
> 6	42	12.8
Time of getting first tattoo		
Less than one year ago	88	26.9
Over a year ago	91	27.8
A few years ago	148	45.3
Education status		
Secondary education	189	57.8
Higher education	112	34.3
Lower secondary education	20	6.1
Vocational	6	1.8
Domicile		
Over 250,000 inhabitants	145	44.3
25–50,000 inhabitants	93	28.5
Less than 25 thousand inhabitants	33	10.1
Rural area	56	17.1
Occupational status		
University students	145	44.3
Worked and studied	119	36.4
Worked	46	14.1
Unemployed	17	5.2

N = 327. Number of tattoos varied from 1 to 40. The maximum number of tattooed areas was = 15

The purpose of the first stage of analysis was to determine whether the number of tattoos was related to one’s mental body representations. The results (see Table 2) suggest that no significant differences exists (*p* > 0.05).

**Table 2:** One-way analysis of variance for the number of tattoos

Group	Single tattoo (*n* = 86)	Two tattoos (*n* = 81)	3 to 5 tattoos (*n* = 86)	More than 5 tattoos (*n* = 74)	Significance of differences	
	*M* (*SD*)	*M* (*SD*)	*M* (*SD*)	*M* *(SD)*	*F*(3, 323)	*p*
Body image	21.09 (*9.29*)	21.11 (*10.39*)	20.41 (*9.24*)	21.26 (*10.78*)	.35	.792
Body schema	23.92 (*6.89*)	23.27 (*7.26*)	24.19 (*6.46*)	24.77 (*6.91*)	1.13	.338
Body sense	22.58 (*6.39*)	22.69 (*6.37*)	22.55 (*5.84*)	22.84 (*7.00*)	.24	.866

*N* = 327

The next stage of statistical data analysis was concerned with differentiating specific types of mental body representations. The k-means cluster analysis (Quick Cluster) was used, which, unlike hierarchical cluster analysis, is suitable for analyzing data sets with a large number of cases. Dividing the group of female respondents into subgroups using the k-averages method provides information on the position of each individual in the group (Marek 1989). The k-means cluster analysis revealed three types (clusters) of body self-representations among the studied group of tattooed women (see Table 3). The analysis was performed on the basis of the results (SBS, SBI, SBA scales) obtained from all women, regardless of the number of tattoos they had. The three-cluster approach seemed appropriate to the task due to statistical nature and content of the analyzed data. The three clusters correspond to differently sized groups with different mean values on SBS, SBI, and SBA scales.

**Table 3:** Cluster analysis for body self-representations

Variables	Integrated body self (a) *n* = 150		Unstable body self (b) *n* = 116		Disordered body self (c) *n* = 60		*F*(2, 324)	*p*
	*M*	*SD*	*M*	*SD*	*M*	*SD*		
Body image	29.68	4.10	17.10	4.84	6.82	4.48	640.27	< .001
Body schema	28.61	4.48	23.06	4.35	14.56	4.96	212.98	< .001
Body sense	27.58	3.50	20.66	3.79	14.36	4.85	274.50	< .001

*p* < .001. Ibs vs Ubs = *p* < .001. Ibs vs Dbs = *p* < .001. Ubs vs Dbs = *p* < .001

## Results

**Fig. 1:**
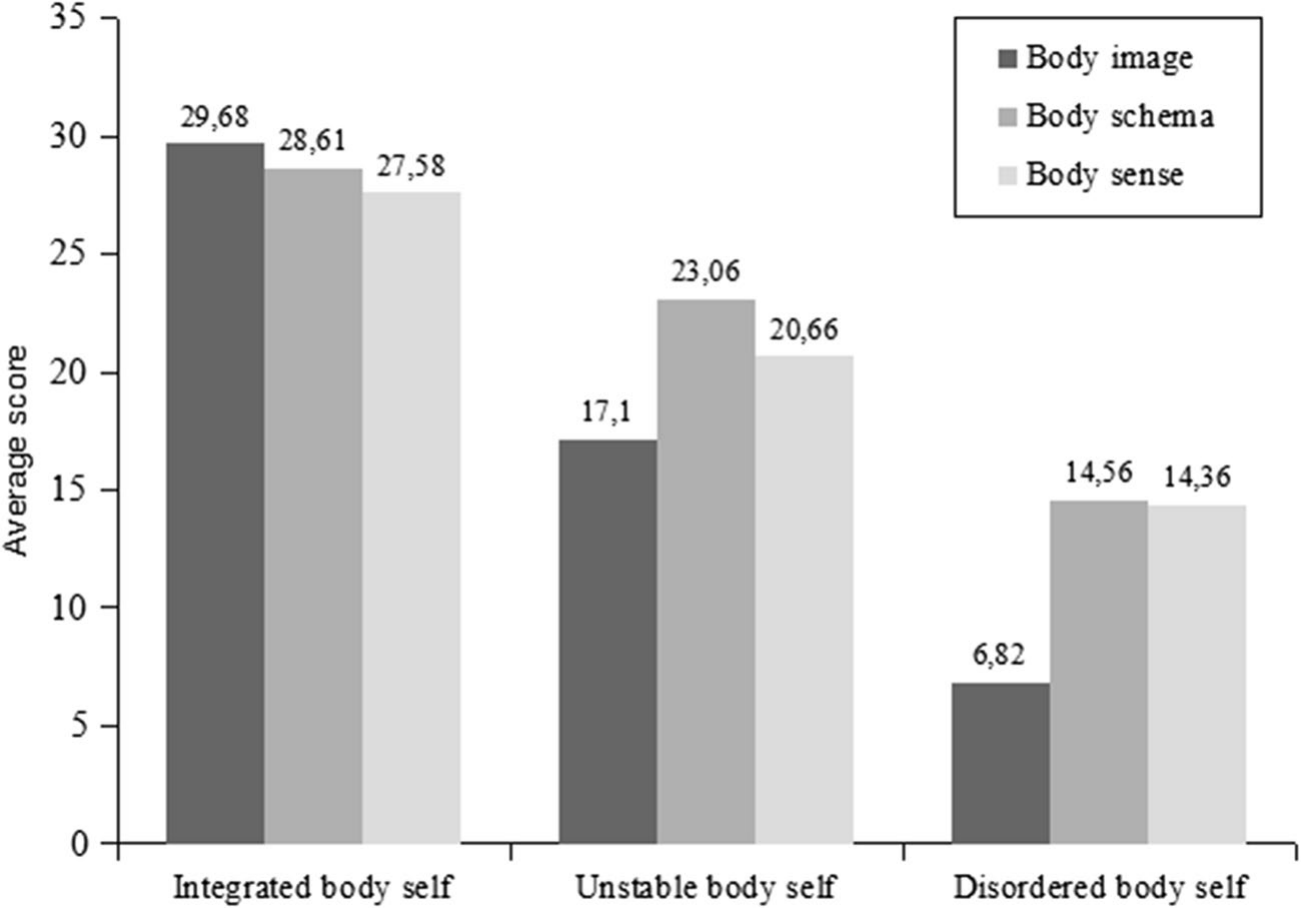
Clustering for body self-representation types in tattooed women

*Integrated body self* (a) characterized the majority (45.26%) of female participants (*N* = 148). This group scored very high on all three body representation scales (*M*_SBI_ = 29.68, *M*_SBS_ = 28.61, *M*_SBA_ = 27.58), with none of the scores being particularly dominant (see Fig. 1).

The second type of body self (*N* = 118, 36.09%), designated as *unstable body self* (b). The participants in this group scored average on all three body representation scales (*M*_SBI_ = 17.10, *M*_SBS_ = 23.06, *M*_SBA_ = 20.60), scoring the lowest on the body image scale and the highest on the body schema scale (see Fig. 1).

The final type of body self, described as *disordered body self* (c), was represented by 61 women participants of the study (18.65%). This group has scored low on all three mental body representation scales (*M*_SBI_ = 6.82, *M*_SBS_ = 14.56, *M*_SBA_ = 14.36), with the body image representation score being significantly lower (see Fig. 1).

Integrated body self (a) vs unstable body self (b) = *p* < 0.001; integrated body self (a) vs disordered body self (c) = *p* < 0.001; and unstable body self vs disordered body self (c) = *p* < 0.001. These results indicate that subjects from cluster Ibs (a) have higher levels of mental representations of the body than subjects from cluster Ubs (b) and cluster Dbs (c), while subjects from cluster Ubs (b) have higher scores than those from cluster Dbs (c) (see Table 3).

Finally, all three distinguished types (clusters) of body self were analyzed in relation to the number of tattoos, number of tattooed areas of the body, and self-esteem of the participants (see Table 4). The analysis showed that a higher unbalance in mental body representation scores corresponded with lower self-esteem in tattooed women. The participants with an *integrated body self* had the highest self-esteem (*M* = 31.45; *SD* = 5.91), while those with a *disordered body self* had the lowest (*M* = 20.85; *SD* = 6.33). In the case of self-assessment, integrated body self (a) vs unstable body self (b) = *p* < 0.001; integrated body self (a) vs disordered body self (c) = *p* < 0.001; and unstable body self vs disordered body self (c) = *p* < 0.001, which indicate that the subjects from cluster Ibs (a) have higher self-esteem than subjects from cluster Ubs (b) and cluster Dbs (c), while subjects from the Ubs (b) cluster are characterized by higher self-esteem than those from Dbs (c).

**Table 4:** Cluster analysis for number of tattoos, number of tattooed areas, and self-esteem

Variables	Integrated body self (a) *n* = 150		Unstable body self (b) *n* = 116		Disordered body self (c) *n* = 60		*F*(2, 324)	*p*
	*M*	*SD*	*M*	*SD*	*M*	*SD*		
Number of tattoos	1.73	.44	1.74	.44	1.74	.44	.01	.99
Number of tattooed body areas	3.06	2.33	3.02	2.50	2.74	2.03	.43	.65
Self-esteem	31.45	5.91	26.43	5.48	20.85	6.33	75.45	< .001

Self-esteem: Ibs vs Ubs = *p* < .001. Ibs vs Dbs = *p* < .001. Ubs vs Dbs = *p* < .001

## Discussion

The aim of this study was to explore the mental body representations of tattooed women during emerging adulthood. The gathered data revealed that the number of tattoos and tattooed body areas are largely unrelated to their mental body representations. This means that the body self-awareness in tattooed women might be treated as a psychological phenomenon whose development does not depend on the external, outward aspects of the tattooed image, but rather on individual person’s internal motivational factors. During our investigation of mental body representations in tattooed women we have discovered three types of them: (1) integrated, (2) unstable, (3) disordered.

## Findings and theory

The first variation of mental body representations in tattooed women was described as the *integrated body self*. This type was found in the largest percentage of participants (approx. 45%). It is associated with a positive body experience, experience of one’s own body as the origin of their agency and self-efficacy, as well as acceptance of one’s own appearance. The gathered data is consistent with findings of Mun et al. (2012), who illustrated that tattooed individuals display a deep connection with their bodies. For women with an integrated body self, tattooing is likely an act of adorning their body, further increasing its attractiveness. The tattoo, thus, is used to articulate the self and one’s individuality in social situations, analogous to other forms of self-expression (e.g., clothing or jewelry). In consequence, for women with an integrated body self, the aesthetic and expressive functions of the tattoo empower their sense of self-worth and perhaps positively impact body identity and personal identity development (Mirucka 2018).

The second type, which we refer to as the *unstable body self*, is associated with distorted mental body representations. It is primarily linked to difficulty in accepting one’s looks. Young females with this type of body experience feel discomfort as they focus their attention on the blemishes in their physical appearance. Periods of relative positive body image are disrupted by fixation on those areas of the body that are perceived as unattractive. The females that belong to this group likely utilize tattoos to conceal the features of their body that fail to meet traditional standards of feminine beauty. Due to issues in building a stable, positive body image, the body experience may become distorted, leading to lower psychophysical integrity. While they can handle body-related emotions rather well, during periods of increased psychological discomfort, these individuals will probably struggle to control their anxiety and shame arising from a negative body self-perception.

The third and final variant of mental body representations found in tattooed women aged 18 to 25 is the *disordered body self.* This type was found in approx. 19% of interviewed women. The participants in this group scored low on all three scales of mental body representations, scoring the lowest on the body image scale. Women with this type of body self-experience significant difficulty in reading internal body sensations and have a distorted sense of control of their own body. They likely struggle with understanding the information originating from within their body and the impact that information has on their emotions. Instead of viewing their body as a valuable instrument of locomotion (Damasio 2000), they perceive it as a handicap which is the source of their overburdening sensations and emotional states. Presumably, the heavily distorted body image of individuals with this type of body self can help us understand the way they regulate their negative emotions. One of the explanations is that they may use tattooing in place of self-injury. The act of tattooing is capable of diverting one’s attention; suppressing “unbearable” emotions through physical pain (Favazza and Rosenthal 1993; Favazza 1998; 2006). Body image is of crucial importance to people with tattoos (Kertzman et al. 2019). Women who exhibit a disordered body self strongly dislike their appearance. They view their own body as unattractive, even “defective,” necessitating serious modification — which is accomplished via tattoos, a permanent alteration of their appearance. The results of this study also correspond to findings of Stirn and Hinz (2008), which showed a connection between body modification (tattoos, piercings) and self-injury. Twenty-seven percent of participants in this study admitted to self-harm behavior during childhood. The abovementioned authors postulated that people with a history of self-harm may use tattoos as a substitute for self-injury. In other words, just as self-injury assists in regulation of emotional strain and management of dissociation, tattoos may adaptively help manage difficult emotional states. This view is supported by Claes et al. (2005) who determined that in individuals suffering from eating disorders, body modifications can help prevent acts of extreme self-injury.

The results of this study show that in tattooed women aged 18 to 25, there’s a strong connection between one’s mental body representations (one of the three types of body self) and their self-esteem. Women with an integrated body self had the highest self-esteem, while those with a disordered body self had very low self-esteem. This reveals that the self-esteem of tattooed individuals is not so much influenced by their tattoos as it is by their mental body representations. The inconclusiveness of various studies of tattooed individuals (Farrow et al. 1991; Kertzman et al. 2019; Pajor et al. 2015; Swami 2011) might have been a product of their noninclusion of the subject of mental body representations (Mirucka 2018).

## Implications from practice

An investigation of cases of disordered body self among tattooed women as well as the disordered body self in women affected by bulimia (Mirucka 2006) suggests that women from both groups exhibit an overwhelming dissatisfaction with their bodies, coupled with negative emotional states of anger, animosity and shame. The intensity of emotions directed at their own body likely activates similar self-aggression behaviors. In women who suffer from eating disorders, body dissatisfaction is externalized via self-aggressive behavior (e.g. overeating followed by purging), while in tattooed women, it is externalized via body modification (self-injury). Some research even suggests that women who suffer from eating disorders experience reduced symptoms after getting a body modification (Claes et al. 2005). It is possible that in their case, the process of tattooing performs a similar function to self-harm, becoming a mechanism of negative emotion regulation, while the tattoo itself serves to obscure one’s body, reducing its social visibility. The above findings may be of great relevance to therapy for people with tattoos and eating disorders. Tattooing may act as a regulator of body-related emotions, thereby assisting in coping with the difficulties experienced by people with a disturbed bodily self.

## Strengths and limitations

The strengths of the present study are a sufficiently large sample of women with tattoos in emerging adulthood and the use of a concept that takes into account mental representations of the body, which provides quite a lot of insight into the corporeality of women performing tattooing acts.

The greatest limitation of our study was the lack of questions about subjects’ BMI and possible psychiatric history which depleted the study of information regarding possible correlations with experiences of body-related disorders and emotional regulation — something worth addressing in the future (Higgins 1987). Other limitations were no calculated sample size, and the small number of subjects with a large number of tattoos. The number of tattoos (Mortensen et al. 2019), the area of the body, and the content presented on the tattoo (Timming and Perrett 2017) may differentiate the subjects in terms of psychological variables.

Other indicators related to physical appearance also seem to be important. The perception of beauty in a specific culture, skin color, hair color, and physical well-being can also affect the experience of one’s bodily self. Variables related to not only external appearance, but also physical as well as mental illnesses, should be controlled in future studies because of their impact on experiencing one’s body.

Another limitation of the study is that the group of men with tattoos was not included. Therefore, it would be worthwhile in future research to explore important differences between the body experience of women and men with tattoos, as well as to test the importance of factors such as self-objectification and internalization of sexism in the occurrence of these differences.

The nature of the research, which used self-report methods, is also characterized by subjectivity in the reporting of experienced states. However, our results indicating the variation in body experience among women with tattoos encourage other studies to be undertaken that allow for more objective measurements, such as galvanic skin response (GSR).

## Conclusion

The results of this study invite a new approach to tattoo research in which the tattoo will be treated as a complex social and psychological phenomenon associated with at least three different types of mental body representations in women during emerging adulthood.

Previous research on the population of people with tattoos did not address the issues of mental body representations, which determine the quality of bodily self-experience, focusing not only on the visual aspect but also on the kinesthetic aspect and on the sensations from the inside of the body. In accordance with the assumption of the integration of bodily and mental experiences, this research, indicating the diversity in the experience of the body, may similarly differentiate people with tattoos in terms of other psychological variables, but above all, the aspect of emotional processing seems to be important.

Emotion processing is a psychological area that involves both the body and the mind, which may indicate that emotion regulation in women (Zamani Zarchi et al. 2020) may proceed differently depending on the type of body experience. Further research will attempt to answer an important clinical question: Do and how do people regulate their emotions with the help of a tattoo? Does the tattoo play an important role in the process of overcoming difficult (traumatic) experiences? Do people whose main goal is to use a tattoo for decorative purposes and self-expression differ significantly in terms of experiencing their body from people who get tattooed because of the desire to express and regulate their emotions?

## Supplementary Information

Below is the link to the electronic supplementary material.

(PNG 124 KB)

(PNG 120 KB)

## Data Availability

Supplemental material for this article is available online.
