# Supplementary figures and images for: Mental body representations of women with tattoos in emerging adulthood — a cluster analysis
Source: Arch Womens Ment Health. 2023 Jun 1;26(4):473–83. doi: 10.1007/s00737-023-01326-z (PMC10333373; doi:10.1007/s00737-023-01326-z)

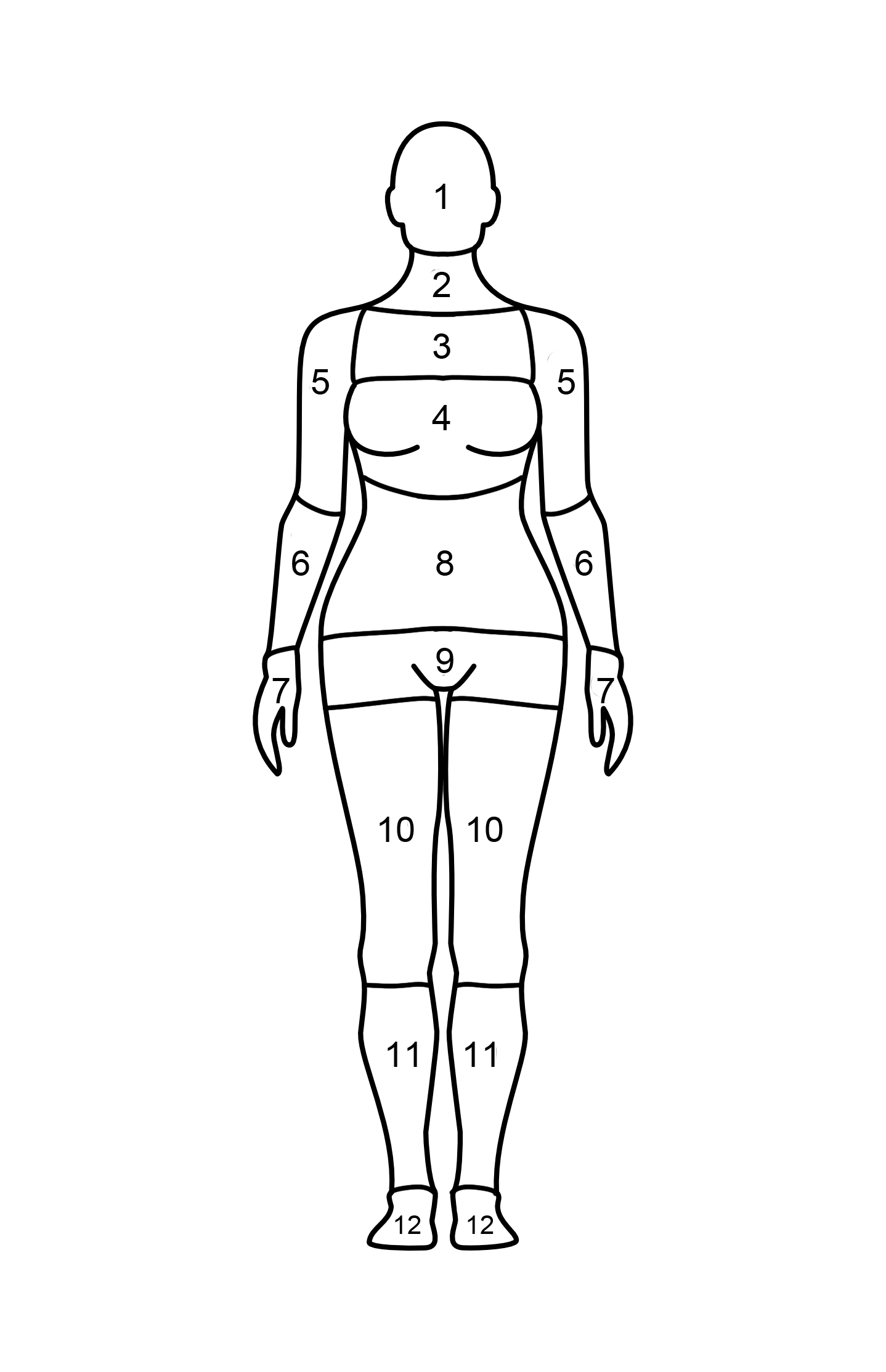

Supplement: Supplementary file 1 — (PNG 124 KB) [file 737_2023_1326_MOESM1_ESM.png]

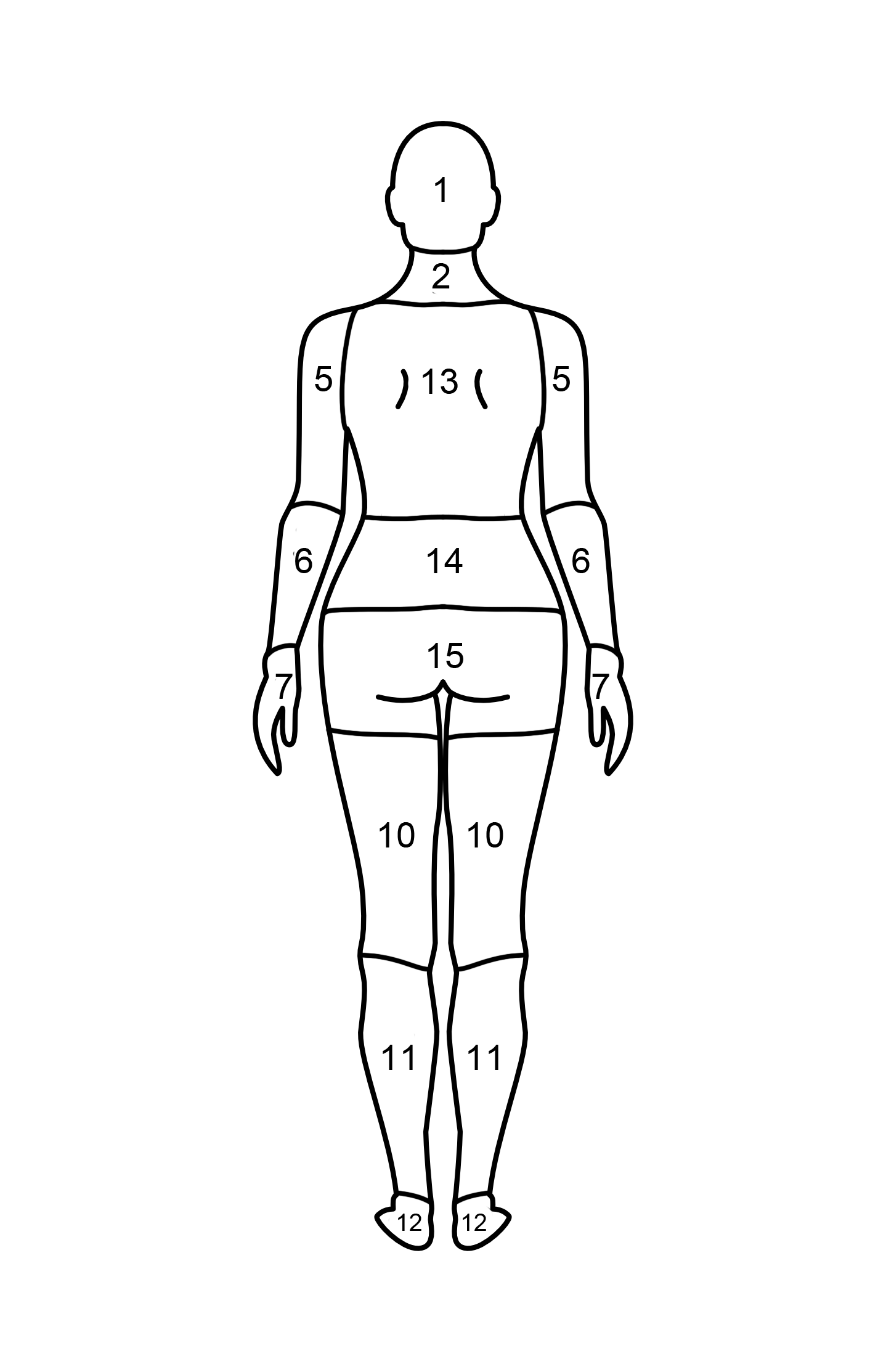

Supplement: Supplementary file 2 — (PNG 120 KB) [file 737_2023_1326_MOESM2_ESM.png]
